# BRD4 PROTAC degrader ARV-825 inhibits T-cell acute lymphoblastic leukemia by targeting 'Undruggable' Myc-pathway genes

**DOI:** 10.1186/s12935-021-01908-w

**Published:** 2021-04-22

**Authors:** Shuiyan Wu, You Jiang, Yi Hong, Xinran Chu, Zimu Zhang, Yanfang Tao, Ziwei Fan, Zhenjiang Bai, Xiaolu Li, Yanling Chen, Zhiheng Li, Xin Ding, Haitao Lv, Xiaoli Du, Su Lin Lim, Yongping Zhang, Saihu Huang, Jun Lu, Jian Pan, Shaoyan Hu

**Affiliations:** 1 Department of Hematology, Children’s Hospital of Soochow University, No.92 Zhongnan Street, SIP, Suzhou, 215003 Jiangsu China; 2 Intensive Care Unit, Children’s Hospital of Soochow University, Suzhou, 215003 China; 3 Department of Pediatrics, Changshu Hospital Affiliated to Nanjing University of Chinese Medicine, Suzhou, 215506 China; 4 Institute of Pediatric Research, Children’s Hospital of Soochow University, No.92 Zhongnan Street, SIP, Suzhou, 215003 China; 5 Department of Neonatology, Children’s Hospital of Soochow University, Suzhou, 215003 China; 6 Department of Cardiology, Children’s Hospital of Soochow University, Suzhou, 215003 China; 7 Division of Human Genetics, Cincinnati Children’s Hospital Medical Center, Cincinnati, OH 45229 USA; 8 Department of Internal Medicine, Saint Michael’s Medical Center, Newark, NJ 07102 USA

**Keywords:** BRD4, ARV-825, T-cell acute lymphoblastic leukemia, Pediatric, C-Myc

## Abstract

**Background:**

T-cell acute lymphoblastic leukemia (T-ALL) is an aggressive disease with a high risk of induction failure and poor outcomes, with relapse due to drug resistance. Recent studies show that bromodomains and extra-terminal (BET) protein inhibitors are promising anti-cancer agents. ARV-825, comprising a BET inhibitor conjugated with cereblon ligand, was recently developed to attenuate the growth of multiple tumors in vitro and in vivo. However, the functional and molecular mechanisms of ARV-825 in T-ALL remain unclear. This study aimed to investigate the therapeutic efficacy and potential mechanism of ARV-825 in T-ALL.

**Methods:**

Expression of the BRD4 were determined in pediatric T-ALL samples and differential gene expression after ARV-825 treatment was explored by RNA-seq and quantitative reverse transcription-polymerase chain reaction. T-ALL cell viability was measured by CCK8 assay after ARV-825 administration. Cell cycle was analyzed by propidium iodide (PI) staining and apoptosis was assessed by Annexin V/PI staining. BRD4, BRD3 and BRD2 proteins were detected by western blot in cells treated with ARV-825. The effect of ARV-825 on T-ALL cells was analyzed in vivo. The functional and molecular pathways involved in ARV-825 treatment of T-ALL were verified by western blot and chromatin immunoprecipitation (ChIP).

**Results:**

BRD4 expression was higher in pediatric T-ALL samples compared with T-cells from healthy donors. High BRD4 expression indicated a poor outcome. ARV-825 suppressed cell proliferation in vitro by arresting the cell cycle and inducing apoptosis, with elevated poly-ADP ribose polymerase and cleaved caspase 3. BRD4, BRD3, and BRD2 were degraded in line with reduced cereblon expression in T-ALL cells. ARV-825 had a lower IC50 in T-ALL cells compared with JQ1, dBET1 and OTX015. ARV-825 perturbed the H3K27Ac-Myc pathway and reduced c-Myc protein levels in T-ALL cells according to RNA-seq and ChIP. In the T-ALL xenograft model, ARV-825 significantly reduced tumor growth and led to the dysregulation of Ki67 and cleaved caspase 3. Moreover, ARV-825 inhibited cell proliferation by depleting BET and c-Myc proteins in vitro and in vivo.

**Conclusions:**

BRD4 indicates a poor prognosis in T-ALL. The BRD4 degrader ARV-825 can effectively suppress the proliferation and promote apoptosis of T-ALL cells via BET protein depletion and c-Myc inhibition, thus providing a new strategy for the treatment of T-ALL.

**Supplementary Information:**

The online version contains supplementary material available at 10.1186/s12935-021-01908-w.

## Background

T-cell acute lymphoblastic leukemia (T-ALL) is serious disease with a high risk of induction failure, with up to 20% of children and 40% of adults relapsing after intensive combination chemotherapy [1]. Relapsed T-ALL is associated with poor outcomes, with an estimated 5-year overall survival of less than 10% [2, 3]. Chemoresistance is major cause of recurrence and refractory T-ALL in both adult and pediatric patients, despite the use of inhibitors targeting the NOTCH1 pathway [3]. There is thus an urgent need to discover new treatment strategies based on the genomic landscape.

The bromodomain and extraterminal (BET) protein family member BRD4 is a transcriptional and epigenetic regulator known to be involved in super-enhancer organization and transcriptional regulation in cancers [4, 5]. Previous studies reported that over expression of BRD4 was linked to tumorigenesis and progression in several solid tumors and some hematologic malignancies [6–9]; however, its role in T-ALL remains unclear. The need for effective treatments for T-ALL combined with the development of small-molecule BET inhibitors thus prompted us to investigate the suitability of BRD4 as a T-ALL drug target.

Several structure- and activity-based BET protein small-molecule inhibitors have been developed, including JQ1 and OTX015. These agents disrupt the binding of BRD4 to acetylated chromatin, and inhibit growth and induce apoptosis in a variety of cancers, including hematologic malignancies and solid tumors [10–13]. However, some studies found that BET inhibitors led to the accumulation of BRD4 protein in cancer cells, which, together with the reversible nature of inhibitor binding, could prevent efficient BRD4 inhibition [14–17]. Using the Proteolysis-targeting Chimera (PROTAC) platform, Lu et al. recently designed a novel chimeric molecule ARV-825, comprising OTX015 conjugated with an E3 ubiquitin ligase cereblon (CRBN), which could efficiently degrade BRD4 [18]. Several studies showed that ARV-825 suppressed proliferation and induced apoptosis more effectively and had longer-lasting effects than BRD4 inhibitors, possibly via the rapid and long-lasting degradation of BRD4 and suppression of downstream targets such as c-Myc [14, 15, 17–19]. Meanwhile, we previously showed that ARV-825 reduced cell growth and increased apoptosis in neuroblastoma and multiple myeloma [16]. We therefore investigated the antitumor activity of ARV-825 targeting BRD4 in T-ALL, with the aim of identifying effective strategies for the treatment of pediatric T-ALL.

## Methods

### Samples

This study was carried out in accordance with The Code of Ethics of the World Medical Association (Declaration of Helsinki) and was approved by the ethics committee of the Children’s Hospital of Soochow University (2020CS009). A total of 46 human diagnostic T-ALL bone marrow samples were collected from participants in the CCLG-2008 protocol [20], and BRD4 mRNA expression was determined by RNA sequencing (RNA-seq). The clinical features of the patients are shown in Additional file 1: Table S1. 16 diagnostic pediatric T-ALL bone marrow samples and 9 healthy donor samples selected by Dynabeads™ CD4 (cat. No. 00737290; Thermo Fisher Scientific, Waltham, MA, USA), according to the manual, were used to detect BRD4 mRNA expression via real-time polymerase chain reaction (PCR). Samples from two patients with primary T-ALL who participated in the CCCG 2015 protocol [21] were collected for cell viability and western blot analysis.

### Cell culture

Human T-ALL cell lines, including Jurkat, CCRF-CEM (CCRF), Molt4, and 6 T-CEM, were obtained from the cell bank of the Chinese Academy of Science and cultured in RPMI medium (Thermo Fisher Scientific) containing 10% fetal bovine serum (Biological Industries, CT, USA), and 1% penicillin–streptomycin (Millipore, Billerica, MA, USA) at 37 °C in a humidified incubator with an atmosphere of 5% CO_2_ and tested routinely for mycoplasma. All T-ALL cell lines were authenticated by short tandem repeat analysis in 2019 and 2020.

### Cell viability assay

T-ALL cells were seeded in 96-well plates at a density of 2 × 10^4^ cells per well and treated continuously with different concentrations of ARV-825. Primary leukemic cells were isolated from bone marrow by Ficoll–Hypaque centrifugation, and then seeded in 96-well plates at a density of 1 × 10^5^ cells per well in culture medium for 7 days. Cells treated with 0.05% dimethyl sulfoxide (DMSO) in complete medium without ARV-825 were used as controls. ARV-825 (Sigma-Aldrich, St. Louis, MO, USA) was dissolved in 100% DMSO to obtain a 10 mM stock solution, aliquoted, and stored at − 20 °C until analysis. After drug treatment for 48 h, cell viability was determined by Cell Counting kit-8 (CCK8) assay (Dojindo Molecular Technologies, Tokyo, Japan) according to the manufacturer’s instructions. Cell proliferation was calculated as a percentage of that in cells in control medium. Each concentration was tested in triplicate and repeated in at least three independent experiments. The half maximal inhibitory concentration (IC50) of ARV-825 was calculated using Graph Prism software 8.3.0 (GraphPad Software Inc., San Diego, CA, USA).

### Lentivirus preparation and infection

Short hairpin RNA (shRNA) targeting CRBN (CCGGGCCCACGAATA GTTGTCATTTCTCGAGAAATGACAACTATTCGTGGGCTTTTTG) constructed in the pLKO.1 lentiviral vector [16] and pLX304-CRBN-V5 vector [22] was a kind gift from Dr. X. Liang (Cancer Science Institute, Singapore). For lentivirus preparation, the envelope plasmid and packaging plasmid were purchased from Addgene (pMD2.G: #12,259; psPAX2: #12,260; Cambridge, MA, USA). pMD2.G, psPAX2 and the transfer plasmid were cotransfected into 293FT cells using polyethylenimine (linear MW 25,000 Da, 5 mg/mL, pH 7.0) (cat. No. 23966–1; Polysciences, Warrington, PA, USA) according to the manufacturer’s instructions. After 6 h, the culture medium was completely replaced with fresh medium. The viral supernatant was harvested at 48 h post-transfection and filtered through a 0.22 μm filter. T-ALL cells were then infected with lentivirus in the presence of 10 μg/mL Polybrene (Sigma-Aldrich) for 24 h. Stable cell lines were selected with puromycin or blasticidin (Sigma-Aldrich).

### RNA-seq and data processing

RNA-seq was carried out according to the protocols suggested by BGI (AppTec, Wuxi, China). First, total RNA was reverse transcribed to cDNA for library construction, and the cDNA library was then sequenced. The raw reads were filtered and clean reads were mapped according to Bowtie2 and HISAT. The gene expression level (as fragments per kilobase of exon model per million reads mapped) was then calculated. For analysis of differential gene expression, raw RNA sequencing data were analyzed using the DAVID Bioinformatics Resources v6.8 online server (https://david.ncifcrf.gov).

T-ALL cells (Jurkat and 6 T-CEM) treated with DMSO (n = 2) or ARV-825 (n = 2) after 48 h were also subjected to RNA-seq. Differentially expressed genes (P < 0.001 and fold-change > 2) were identified using limma analysis. Gene set enrichment analysis (GSEA) using the Java GSEA Desktop Application (http://www.broadinstitute.org/gsea/) identified a set of genes that was subsequently subjected to Cytoscape analysis (http://www.cytoscape.org/) to detect cellular pathways influencing the level of ARV-825-induced cellular death.

### RNA preparation and real-time PCR expression analysis

Total RNA was extracted from cell pellets using TRIzol^®^ reagent (Invitrogen, CA, USA), according to the manufacturer’s protocol. For cDNA synthesis, 2 µg of total RNA was converted to cDNA using a High-Capacity cDNA Reverse Transcription Kit (Applied Biosystems, CA, USA). Quantitative real-time PCR analysis was carried out using LightCycler^®^ 480 SYBR Green I Master mix (cat. No. 04707516001; Roche, Penzberg, Germany) with a LightCycler 480 Real Time System (Roche), according to the manufacturer’s protocol. mRNA expression levels were calculated using the Ct method with actin expression as an internal reference. The real-time PCR primers were as follows: β-actin: forward, 5′-TTGCT GACAGGATGCAGAAGGAGA-3′ and reverse, 5′-ACTCCTGCTTGCTGA TCCACATCT-3′; BRD4 sense, 5′-ACCTCCAACCCTAACAAGCC-3′ and antisense, 5′-TTTCCATAGTGTCTTGAGCACC-3′.

### Cell cycle analysis

T-ALL cells were trypsinized, washed, and fixed in 70% ethanol at 4 °C overnight. The cells were then washed with cold phosphate-buffered saline (PBS) and resuspended in 0.5 mL of PI/RNase Staining Buffer (cat. No. 550825; BD Pharmingen™, San Diego, CA, USA), followed by incubation at room temperature for 15 min. The cells were then subjected to flow cytometry using a Beckman Gallios™ Flow Cytometer (Beckman, Krefeld, Germany) and the cell cycle was analyzed with MultiCycle AV DNA analysis software (Verity Software House, Topsham, ME, USA).

### Cell apoptosis assay

Cell apoptosis was determined as described previously [23]. Briefly, T-ALL cells were incubated with ARV-825 at the indicated concentrations for 48 h, harvested and washed with cold PBS, suspended in 1 × binding buffer, and stained with fluorescein isothiocyanate (FITC)-Annexin V antibody and PI solution using an FITC-Annexin V apoptosis kit (cat. No.556420; BD Biosciences, Franklin Lakes, NJ, USA), according to the manufacturer’s instructions. Cell apoptosis was analyzed by flow cytometry (Beckman Gallios™ Flow Cytometer; Beckman).

### Western blot analysis

Western blot analysis was conducted as described previously [23] using the following primary antibodies: BRD2 (cat. No. 5848 s; 1:1000; Cell Signaling Technology, Boston, MA, USA), BRD3 (cat. No. 11859-1-AP; 1:1000; Proteintech, Chicago, IL, USA), BRD4 (cat. No. 13440 s; 1:1000; Cell Signaling Technology), CRBN (cat. No. HPA045910; 1:1000; Sigma-Aldrich), c-Myc (cat. No. 9402; 1:1000; Cell Signaling Technology), cleaved caspase 3 (cat. No. 9664; 1:1000; Cell Signaling Technology), and PARP (cat. No. 9542; 1:1000; Cell Signaling Technology), with glyceraldehyde 3-phosphate dehydrogenase (GAPDH) (cat. No. MA3374; 1:1000; Millipore) as a reference protein. Peroxidase-conjugated Affiniure goat anti-rabbit IgG (H + L) (cat.111-035-003; 1:5000) and goat anti-mouse IgG (H + L) (cat. No. 115-035-003; 1:5000) secondary antibodies were purchased from Jackson ImmunoResearch Laboratories, Inc. (West Grove, PA, USA). ImageJ software was used for band quantification. The values obtained were normalized to 0.1% DMSO vehicle control. Then, protein levels were determined using a GAPDH antibody for normalization. DC50 value was determined by assuming a linear model between the two data points across 50% protein level mark. To define the role of proteasome, MG132 (cat. No. 474787, Sigma-Aldrich, St. Louis, MO, USA) was applied in the inhibition of proteasome activity. After treatment with 1 μM ARV-825 and various concentration of MG132 at 24 h, cells were collected and BRD2, BRD3 and BRD4 protein were determined by Western blot.

### Chromatin immunoprecipitation (ChIP)

ChIP was performed as described previously [24]. Briefly, 3–5 × 10^7^ cells were crosslinked with 1% formaldehyde for 10 min and neutralized with 1.25 M glycine for 5 min at room temperature. Fixed cells were harvested, lysed, and sonicated using a Bioruptor (Diagenode, Liège, Belgium). Sonicated chromatin was incubated with anti-histone H3 (acetyl K27) antibody (cat. No. ab4729; Abcam, Cambridge, UK) overnight at 4 °C. DNA was eluted and purified using a QIAquick PCR purification kit (cat. No. 208106; Qiagen, Hilden, Germany). Samples were sequenced on an Illumina HiSeq platform (BGI, Wuhan, China) Ad.

### In vivo xenografts

All animal procedures in this study were approved and licensed by the Animal Care and Use Committee at Children’s hospital of Soochow University (CAM-SU-AP#:JP-2018-1). Nude mice were obtained from Lingc hang BioTech Co., Ltd. (Shanghai, China). Four-week-old female nude mice (n = 5 per group) were injected subcutaneously in the frontier flank with 5 × 10^7^ CCRF cells. Two weeks later, either 10 mg/kg of ARV-825 or vehicle alone (5% Kolliphor^®^HS15) were given intraperitoneally every day. Subcutaneous tumor size was monitored using calipers every 2 days. Tumor volume was calculated according to the formula (width × width × length)/2. Mice euthanized were inhalation of CO_2_ for 5 min and the CO_2_ exposure used a gradual fill method with a displacement rate from 30 to 70% of the chamber volume/min. The immunohistochemistry staining was performed as previously described [25]. The primary antibody against cleaved-caspase 3 (cat: No. GB11009-1, 1:300, Servicebio, Boston, MA, USA) and Ki67 (cat: No. ab15580, 1:300, Abcam, Cambridge, UK) were used according to the manufacturer's recommendations.

## Statistical analysis

All experiments were performed independently at least three times. Statistical analysis was performed using SPSS software version 17.0 (IBM Corporation, NY, USA). Survival analysis was performed by Kaplan–Meier estimates with log-rank tests. Percentages of cell apoptosis, BRD4 mRNA level, and cell viability were compared using Student’s *t*-tests. Normally distributed measured data were expressed as mean ± standard deviation. Differences between two groups were compared using *t*-tests, χ^2^ tests, or the exact probability method. Non-normally distributed data were expressed as quartiles and compared using Mann–Whitney U tests. All tests were two-tailed and P < 0.05 was considered statistically significant.

## Results

### BRD4 was overexpressed in T-ALL patients and associated with poor prognosis

**Fig. 1:**
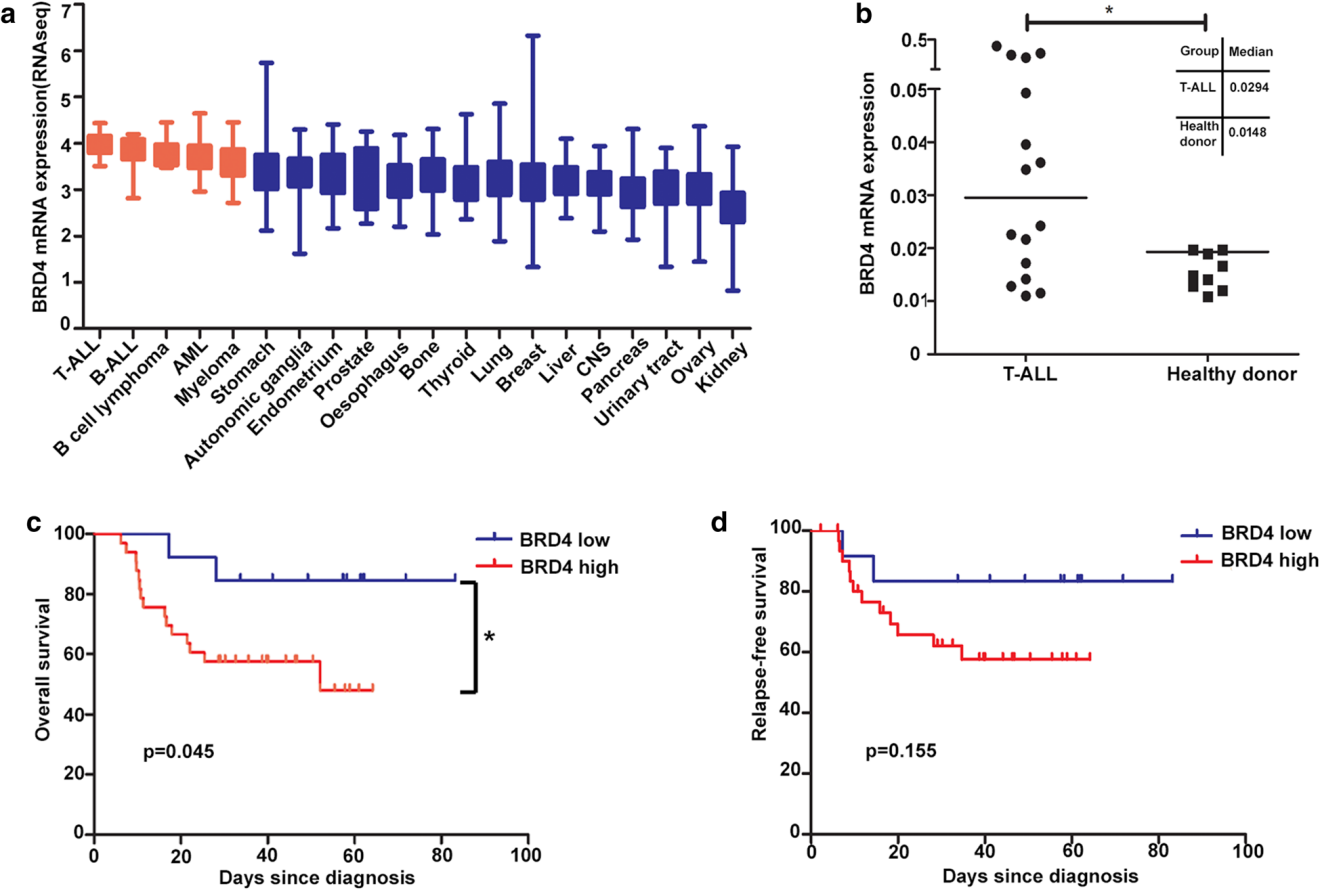
BRD4 is potentially good target of T-ALL. **a** BRD4 mRNA expression level in a broad range of cancer cells (generated from Broad Cancer Cell Line Encyclopedia: https://portals.broadinstitute.org/ccle). **b** BRD4 expression was higher in T-ALL patients (n = 16) than normal T cell samples (n = 9) (p = 0.028). BRD4 expression was detected by real-time quantitative reverse transcription polymerase chain reaction (RT-PCR) and relative expression was represented using comparative CT[2 (−ΔCT)]. **c**, **d** Overall survival and relapse-free survival curve using RNA-seq data of 46 diagnostic T-ALL patients. First quartile with BRD4 mRNA expression was used as a cutoff point for categorized as high or low expression. Overall survival analysis showed that high BRD4 expression was associated with unfavorable outcomes in pediatric T-ALL patients. Relapse-free survival analysis showed an obvious tendency towards higher relapse-free survival in patients with higher BRD4 expression

The Cancer Cell Line Encyclopedia (CCLE; https://portals.broadinstitute.org/ccle) includes BRD4 mRNA expression profiles for different types of cancer cell lines, and showed that BRD4 was highly expressed with no specificity for distinct cancers (Fig. 1a). BRD4 mRNA expression levels in pediatric samples and healthy donors were detected by qPCR. BRD4 expression was increased in T-ALL cells compared with healthy donors (Fig. 1b). To determine the potential utility of targeting BRD4 for the treatment of T-ALL, we analyzed BRD4 mRNA expression via RNA-seq in 46 T-ALL samples. The clinical and molecular characteristics of the patients are shown in Table 1. Reads per kilobase per million mapped reads-normalized gene expression data were used to evaluate the prognostic significance of BRD4 and the association between BRD4, and overall survival of T-ALL patients was also analyzed. BRD4 expression was not related to clinical characteristics, including age, sex, initial bone marrow blasts, white blood cell count, and prednisone response (Table 1). However, high BRD4 expression was associated with unfavorable outcomes in pediatric T-ALL patients (Table 1 and Fig. 1c). There was no significant difference in relapse-free survival between the two groups, but there was an obvious tendency towards lower relapse-free survival in patients with higher BRD4 expression (Fig. 1d). Additionally, multiple logistic regression analyses identified prednisone response as an independent factor affecting survival in pediatric T-ALL patients (P = 0.014) (Table 2), while BRD4 expression was almost a significant independent risk factor for survival (P = 0.064). These results suggest that BRD4 might be a potential therapeutic target for pediatric T-ALL.

**Table 1:** Clinical and molecular characteristics of patients with pediatric acute T-ALL with respect to BRD4 expression

	High BRD4 expression	Low BRD4 expression	*P-Value*
Gender, n (%)			0.409
Male	26 (57)	12 (26)	
Female	7 (15)	1 (2)	
Age at diagnosis,years,median (range)	7.6 (2.3–11.6)	7.9 (1.2–12.2)	0.232
Hemoglobin, g/L, median (range)	102 (54–152)	105 (49–115)	0.518
Platelet, (× 10^9^/L), median (range)	78 (10–263)	65 (30–206)	0.634
Initial BM blast (%)	87 (24–100)	92 (71–97)	0.191
Initial WBC, (× 10^9^/L), n (%)			1.000
< 100	14 (31)	5 (11)	
≥ 100	19 (4)	8 (17)	
Prednisone Response, n (%)			0.738
Good	12 (26)	6 (13)	
Poor	21 (46)	7 (15)	
Day 15th BM, n (%)			0.675
M1 + M2	20 (43)	7 (15)	
M3	13 (28)	6 (14)	
D33th BM blast, n (%)			1.000
M1	29 (63)	11 (24)	
M2	4 (9)	2 (4)	
Week 12th BM blast, n (%)			0.548
M1	30 (65)	13 (28)	
M2	3 (7)	0 (0)	
Risk group, n (%)			0.385
Intermediate risk	29 (63)	10 (22)	
High risk	4 (9)	3 (6)	
Relapse, n (%)			
Yes	12 (26)	2 (4)	0.286
No	21 (46)	11 (24)	
Survival times			
5-years OS, cumulative survival	0.480 ± 0.08	0.846 ± 0.100	0.045
5-years RFS,cumulative survival	0.577 ± 0.094	0.833 ± 0.108	0.155

**Table 2:** Multiple logistic regression analyses of clinical factors associated with overall survival (OS)

	OS				
	B	SE	Exp (B)	95%CI	P
BRD4 expression	1.411	0.762	4.101	0.921–18.258	0.064
Age	0.069	0.676	1.071	0.285–4.030	0.919
WBC (≥ 100)	− 0.742	0.563	0.476	0.158–1.436	0.188
Prednisone Response	1.869	0.757	6.481	1.470–28.574	0.014
D15th BM blast	0.023	0.333	1.023	0.533–1.965	0.945
Gender	0.120	0.661	1.128	0.309–4.117	0.856

### ARV-825 has superior cytotoxicity in T-ALL via promoting apoptosis

**Fig. 2:**
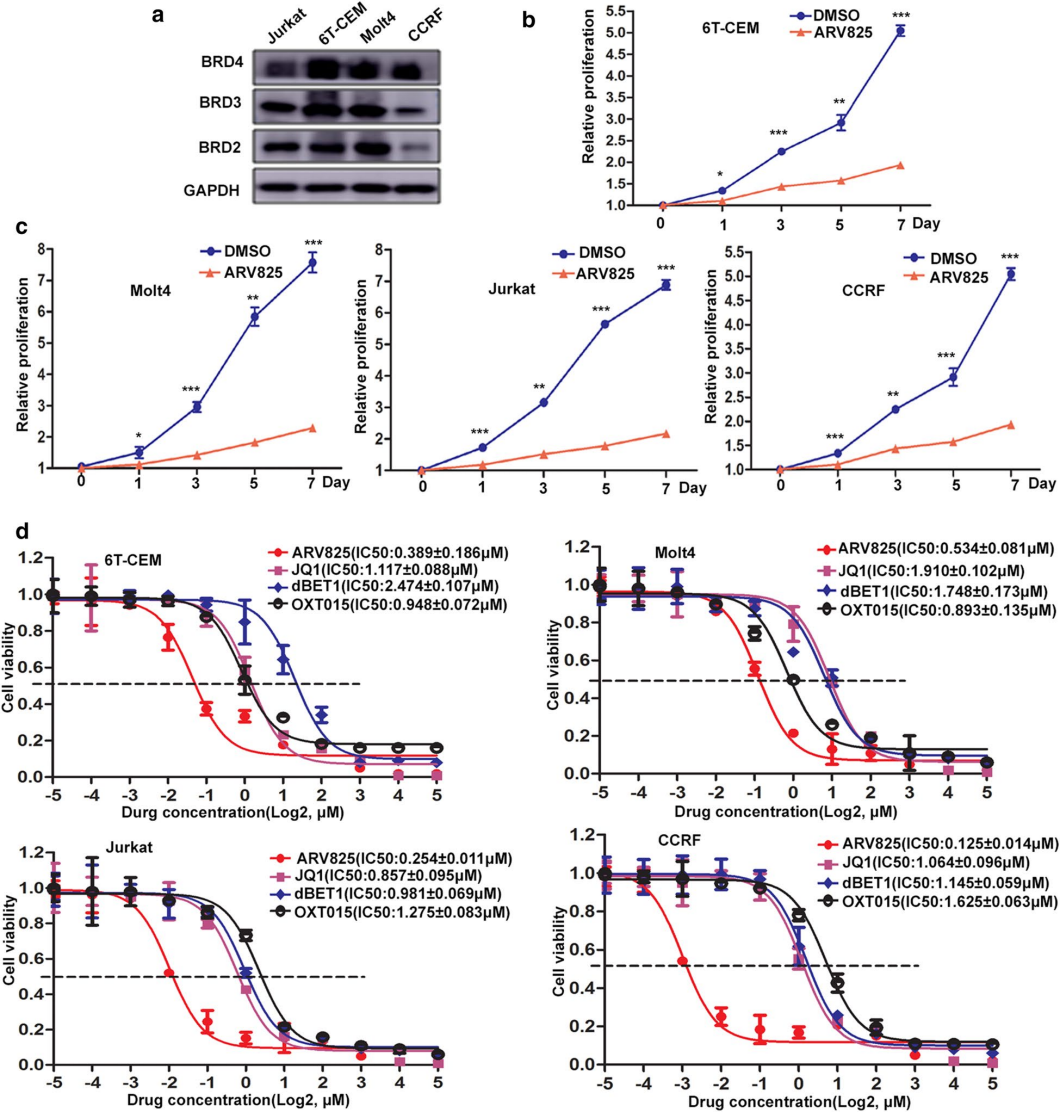
ARV-825 restrained the proliferation of T-ALL cell lines. **a** The basal BET protein level analysis in T-ALL cells, 6 T-CEM, Molt4, Jurkat and CCRF. **b**, **c** The cell growth curve of 6 T-CEM, Molt4, Jurkat and CCRF cells after treatment with DMSO or ARV-825 for 7 days. **d** Drug sensitivity assay of 6 T-CEM, Molt4, Jurkat and CCRF cell lines after treatment with gradient concentrations of ARV-825, JQ1, dBET1 and OTX015 for 48 h

**Fig. 3:**
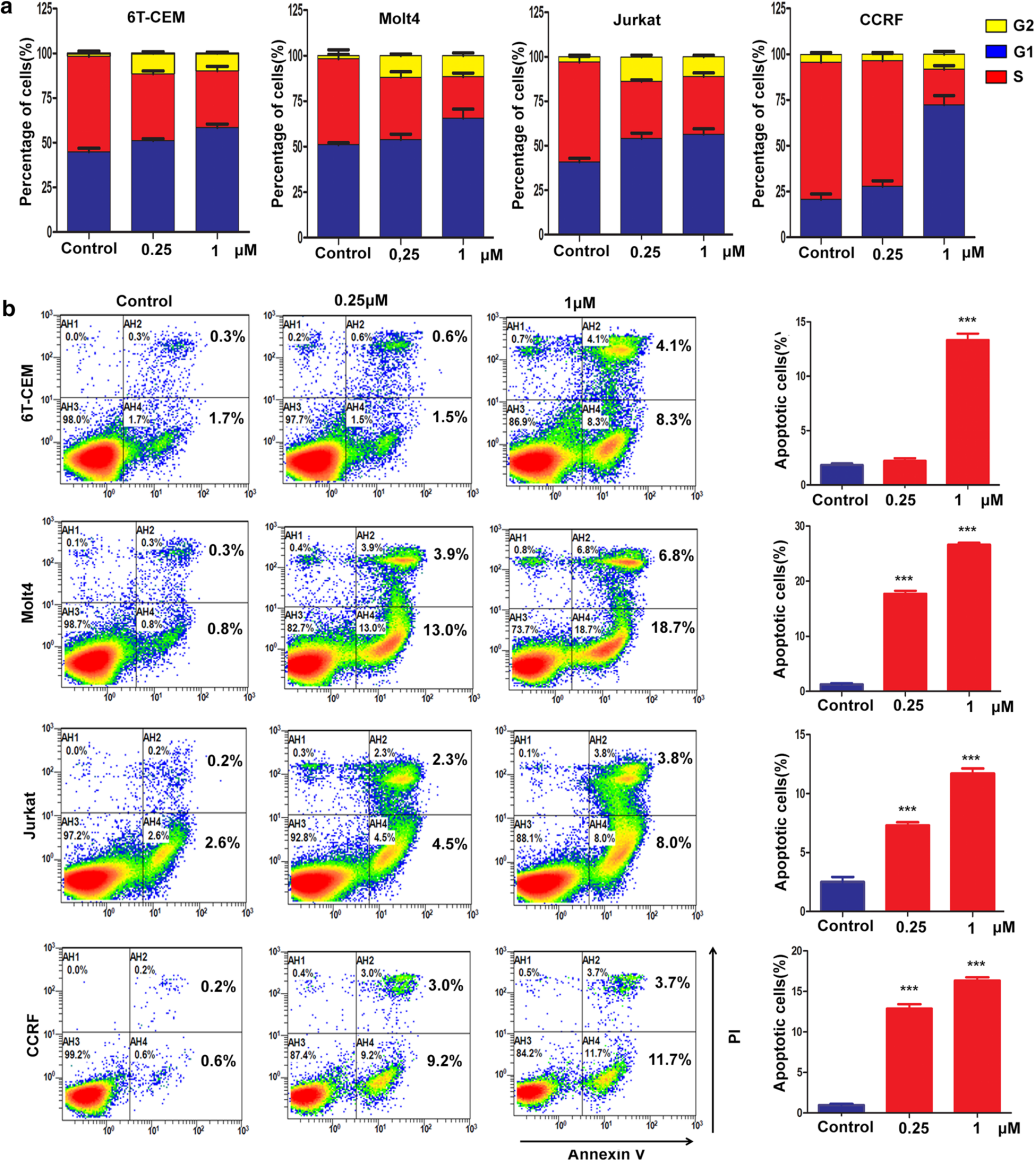
ARV-825 blocked cell cycle and promoted apoptosis of T-ALL cells. **a** PI-labeled cell cycle of 6 T-CEM, Molt4, Jurkat and CCRF cells were analyzed after treatment with DMSO or ARV-825 (1 μM) for 48 h. T-ALL cells were distributed in G1/S phase and the cell population in G1 phase increased dramatically after treatment with ARV-825. **b** Annexin V and PI-labeled cell apoptosis of 6 T-CEM, Molt4, Jurkat and CCRF cells analyzed by flow cytometry after DMSO or ARV-825 (1 μM) treatment for 48 h. The apoptotic rates of T-ALL cell lines were significantly increased after ARV-825 treatment, especially CCRF, Jurkat, and Molt4 cells

**Fig. 4:**
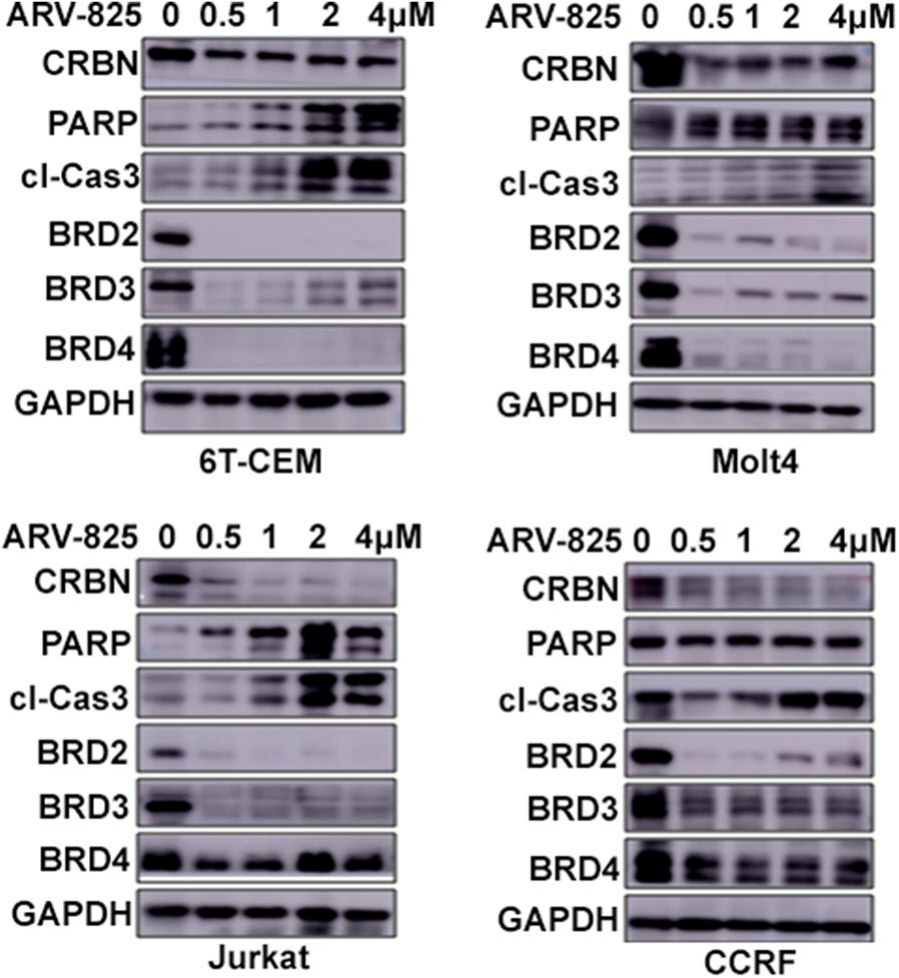
ARV-825 evicts BET protein expression in T-ALL cells. Western blot analysis showed that ARV-825 induced BET proteins degradation, and caspase3 cleavage increase in 6 T-CEM, Molt4, CCRF and Jurkat cells. PARP was increase in 3 of 4 cell lines with concentration-dependent manner

The protein levels of BRD2, BRD3, and BRD4 are shown in Fig. 2a. The BET family members were universally expressed in T-ALL cells. We further explored the cytotoxic effects of ARV-825 in T-ALL cells by examining the effects of 1 μM ARV-825 for 7 days on the viability of Jurkat, CCRF, Molt4, and 6 T-CEM cell lines. ARV-825 significantly reduced the viability of all the tested cell lines in a time-dependent manner (Fig. 2b, c). We evaluated the cytotoxic activity of ARV-825 in T-ALL cell lines by treating them with increasing doses of ARV-825, JQ1, dBET1 OTX015 and for 48 h. T-ALL cell viability was reduced in a dose-dependent manner by treatment with these agents, as shown by CCK8 assay (Fig. 2d), with ARV-825 having a superior cytotoxic effect to JQ1, dBET1 and OTX015 in T-ALL cell lines. We also examined the effects of the cell cycle by staining with PI. Most T-ALL cells were distributed in G1/S phase, but the cell population in G1 phase increased dramatically after treatment with ARV-825 for 48 h (Fig. 3a), suggesting that ARV-825 restrained T-ALL cell proliferation by blocking the cell cycle. Moreover, ARV-825 for 48 h increased the apoptotic ratesof T-ALL cell lines, especially CCRF, Jurkat, and Molt4 cells (Fig. 3b). We also assessed the expression levels of the apoptotic proteins cleaved caspase 3 and PARP by western blot. Cleaved caspase 3 was markedly upregulated in all treated cells and PARP was increased in most of ARV825-treated T-ALL cells, except CCRF (Fig. 4). Collectively, these data suggest that ARV-825 exerted a stronger anti-proliferative effect than other BET inhibitors in T-ALL cell lines.

### ARV-825 evicts BET protein expression in T-ALL cells

ARV-825 was designed by PROTAC technology to selectively degrade target proteins via the ubiquitin proteasome system. We therefore analyzed BET protein expression following ARV-825 treatment in T-ALL cells. Western blotting showed almost complete BRD4 protein degradation in T-ALL cell lines treated with ARV-825 (Fig. 4). In addition to BRD4, ARV-825 also reduced BRD2 and BRD3 protein expression levels. Moreover, ARV-825 is more potent in degrading BRD2 than BRD4 in Jurkat and CCRF cells (Fig. 4). These data suggest ARV-825 downregulated BET protein expression in T-ALL cells.

We next decided to assess the concentration-dependent activity of ARV-825 in T-ALL cells at 48 h after treatments with six different concentrations. Dose-dependent degradation profiles of ARV-825 at the 48 h time-point gave half-degrading concentrations (DC_50_) of 23.12 nM, 16.41 nM and 25.64 nM against BRD2, BRD3 and BRD4 for 6 T-CEM, and that were 13.55 nM, 5.38 nM and 4.75 nM for Molt4, respectively. The DC_50_ values of BRD2, BRD3 and BRD4 proteins were almost around 5 nM for Jurkat. While a higher concentration of ARV-825 (BRD2:34.28 nM; BRD3:29.72 nM; BRD4:225.42 nM) were seen to degrade BRD2, BRD3 and BRD4 protein for CCRF compared to the other three cells (Additional file 2: Figure S1). Together, this data qualified PROTAC ARV-825 as a potent BET proteins degrader.

### CRBN was a powerful helper for ARV-825

**Fig. 5:**
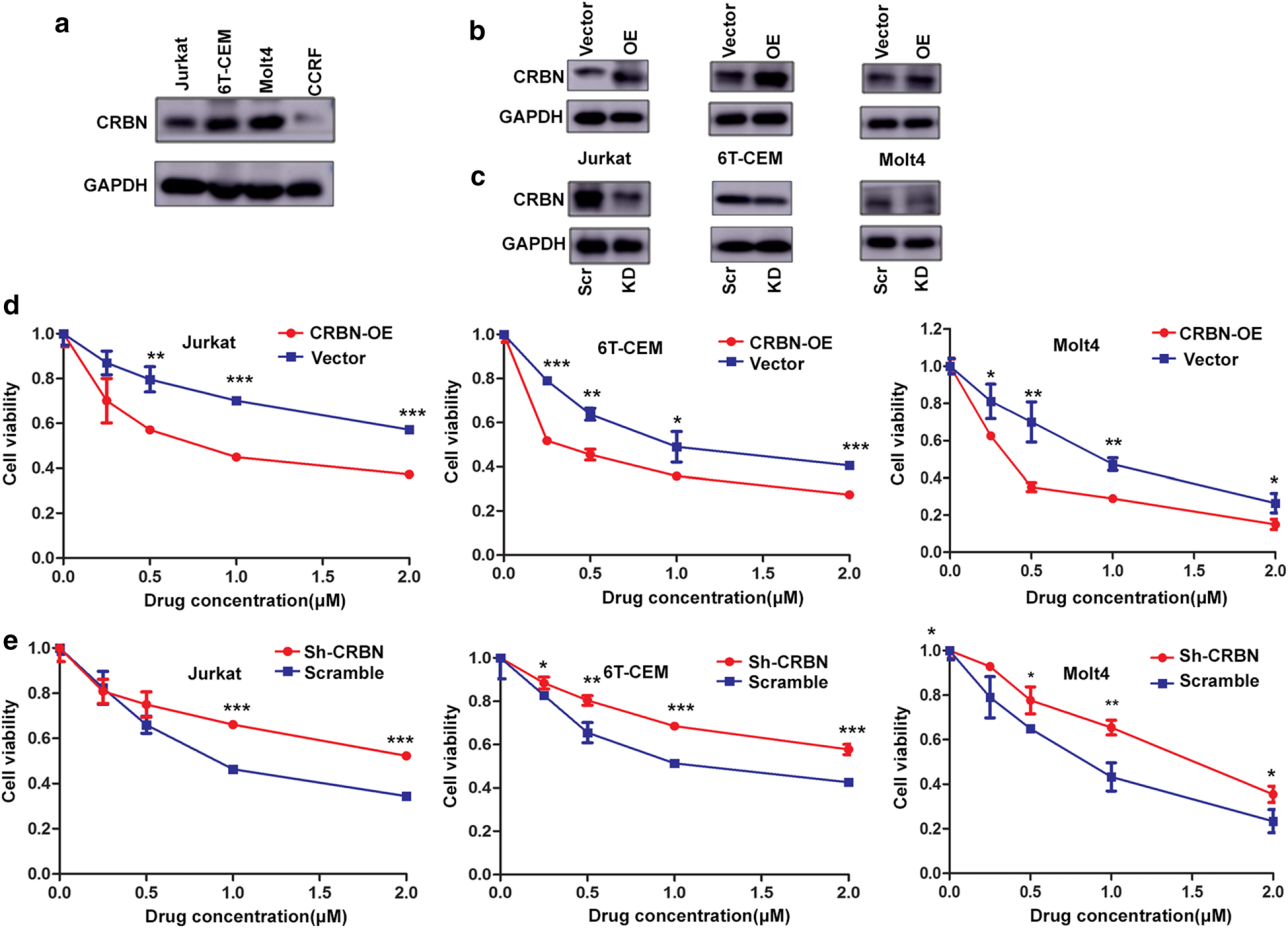
Cereblon was a powerful helper for ARV-825 in T-ALL cells. **a** Western blot analysis of CRBN protein expression in T-ALL cells. **b** Overexpressing CRBN in 6 T-CEM, Molt4 and Jurkat cells with pLX304-CRBN-V5 lentivirus at 5 days. OE: overexpression. **c** Knockdown of CRBN expression by sh-CRBN lentivirus at 5 days in 6 T-CEM, Molt4 and Jurkat cells. Scr: Scramble; KD: knockdown. **d** Comparison of sensitivity to ARV-825 between cells overexpressing CRBN and cells transfected by empty vector alone in 6 T-CEM, Molt4 and Jurkat cells. The results showed excess CRBN reduced the IC50 of ARV-825 in these cells. OE: overexpression. **e** Sensitivity to ARV-825 of cells transfected with sh-CRBN and sh-scramble in 6 T-CEM, Molt4 and Jurkat cells showed that downregulated CRBN increased the IC50 of ARV-825 in these cells

**Fig. 6:**
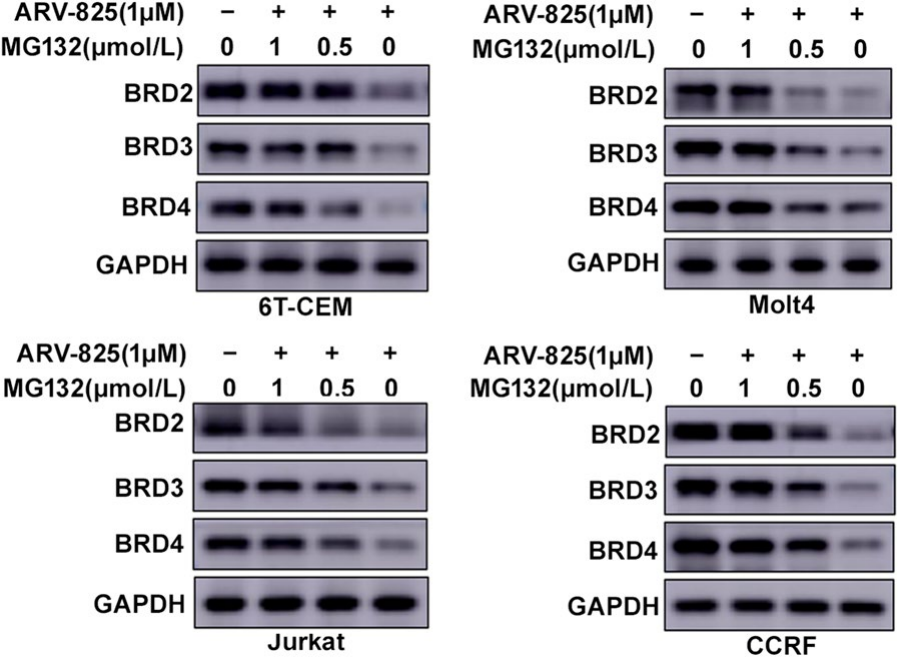
BRDs protein degradation is progeasome-dependent. 6 T-CEM, Molt4, Jurkat and CCRF cells were treated with 1 μM ARV-825 and different concentration of MG132. After treatment with 24 h, BRD2, BRD3 and BRD4 protein were presented by Western blot

We examined the expression of CRBN in T-ALL cell lines (Fig. 5a). CRBN expression levels were lower in CCRF cells compared with other three cell lines, and CRBN-overexpression was not studied in this cell line due to difficulties in transfection. However, we successfully transfected CRBN-overexpression and CRBN-knockdown vectors into Jurkat, 6 T-CEM, and Molt4 cells and verified the expression by western bolt (Fig. 5b, c). ARV-825 comprises OTX015 and a CRBN-recruiting moiety connected by a linker. To confirm that BRD4 degradation induced by ARV-825 was mediated by CRBN, we treated T-ALL cells with ARV-825 in the presence of excess and depleted CRBN. Excess CRBN reduced the IC50 of ARV-825 in 6 T-CEM, Jurkat and Molt4 cells (Fig. 5d), while downregulated CRBN increased the IC50 of ARV-825 in these cells (Fig. 5e). To define the role of proteasome in ARV-825-induced BRDs degradation, proteasome activity was assessed using proteasome inhibitor MG132, which was widely applied in the inhibition of proteasome activity. As anticipated, the results showed a dose-dependent increase in BRDs protein by blocking the proteasome with MG132 (Fig. 6). Taken together, these data demonstrate that ARV-825 caused growth inhibition via a CRBN-mediated mechanism.

### ARV-825 reduces c-Myc expression in T-ALL cells

**Fig. 7:**
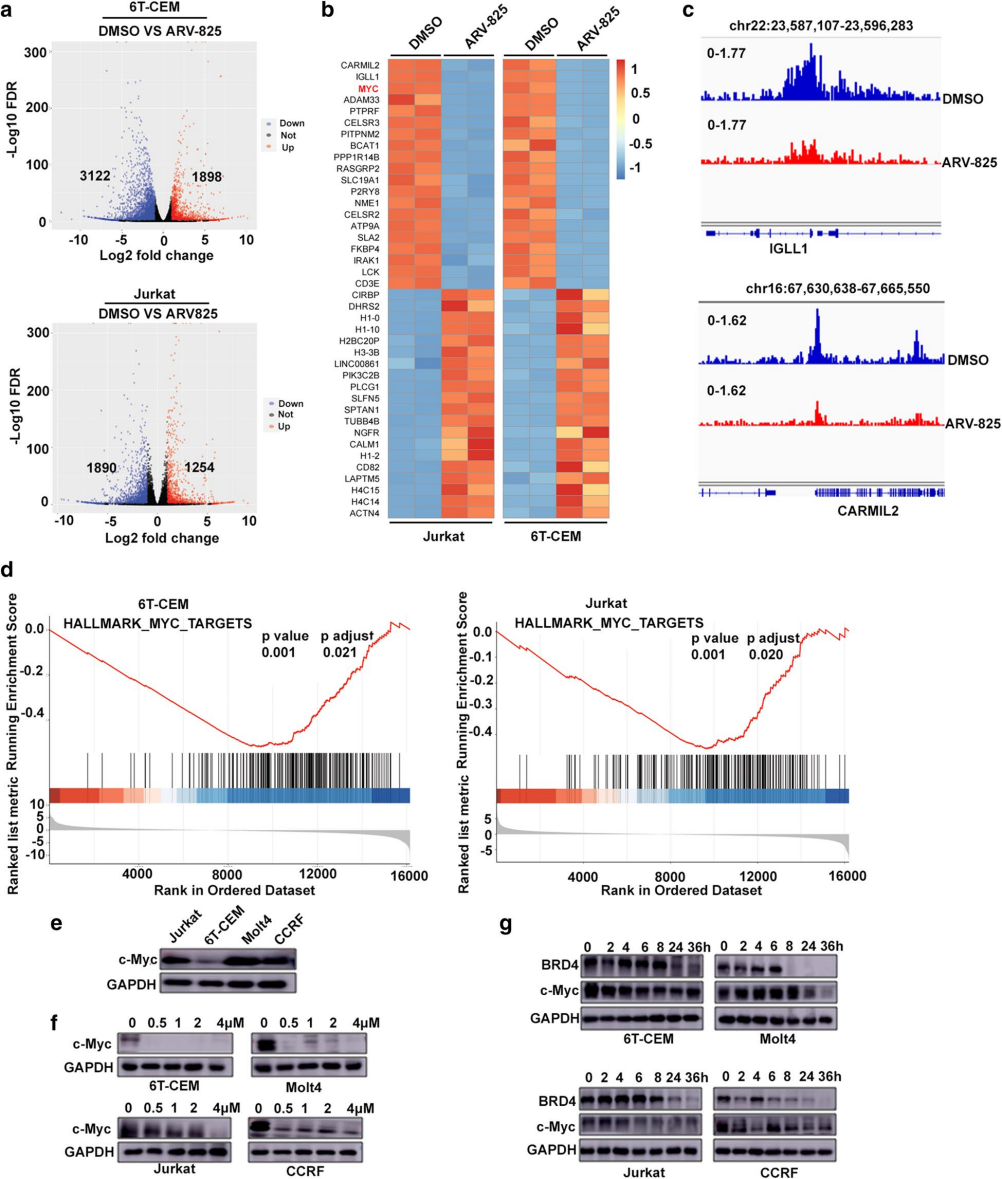
ARV-825 reduces c-Myc expression in T-ALL cells. **a** Volcano plot of RNAseq analysis of gene expression changes in 6 T-CEM and Jurkat cells after ARV-825 treatment. Genes highlighted in red form the up-fold changes, blue indicates down regulated genes and black on behalf of unchanging genes. **b** Heat-map view of the top differentially expressed genes with 20 upregulated and 20 downregulated in 6 T-CEM and Jurkat cells treatment with 1 μM ARV-825 for 48 h (log2FC > 1, P < 0.05). Downregulated genes belong to multiple pro-survival pathways including c-Myc targets. Each column represents a different sample and each row represents a single gene. Color changes within a row indicate expression levels relative to the average of the same population. Red indicates upregulation and blue indicates downregulation. **c** ChIP-seq analysis showed H3K27Ac signal density down regulated with ARV-825 treatment in 6 T-CEM cells. Downregulated genes, IGLL1 and CARMIL2, were confirmed by ChIP-seq analysis in the same T-ALL cells. Results showed that H3K27Ac modification was inhibited significantly with ARV-825 treatment. **d** GSEA plots showing the enrichment of genes in HALLMARK_MYC_TARGETS gene set in RNA-Seq following ARV-825 treatment in 6 T-CEM and Jurkat cells. **e** Western blot analysis of c-Myc expression in 4 T-ALL cells. **f** Western blot analysis showed c-Myc protein was down regulated by treatment with ARV-825 at different concentration in 4 T-ALL cells. **g** Western blot analysis showed c-Myc protein was downregulated by treatment with 1 μM ARV-825 at different times in 4 T-ALL cells

We explored the potential molecular mechanism responsible for the proapoptosis effect of ARV-825 in T-ALL using RNA-seq to identify transcriptionally regulated genes. A total of 1890 genes were downregulated and 1254 were upregulated in Jurkat cells treated with ARV-825 for 48 h compared with the DMSO control group. In 6 T-CEM cells, 3122 genes were downregulated and 1898 were upregulated compared with the control group (Fig. 7a). We screened for genes with fold-change of log2FC > 1 and adjusted P < 0.05, and selected the top 20 downregulated and upregulated genes (Fig. 7b). ARV-825 markedly downregulated *CARMIL2*, *IGLL1*, *MYC*, *ADAM33*, and *PTPRF*, and upregulated histones including *H1-0*, *H1-10*, *H2BC20P*, *H3-3B*, and *H1-2*. ChIP-seq confirmed that ARV-825 downregulated *CARMIL2* and *IGLL1* through disturbing H3K27Ac (Fig. 7c). GSEA plot confirmed that most differentially expressed genes involved in the c-Myc signaling pathway were downregulated (Additional file 3: Table S2 and Additional file 4: Table S3), because their running enrichment score was positive (Fig. 7d). The basal c-Myc expression status of T-ALL cells is shown in Fig. 7e. As expected, c-Myc protein expression was dramatically decreased by BET depletion by ARV-825 in all four T-ALL cells, in dose- and time-dependent manners (Fig. 7f, g). These observations revealed that ARV-825 perturbed BRD4-H3K27Ac mediated c-Myc transcription, leading to reduced c-Myc protein expression.

### ARV-825 has potent antitumor effect in primary T-ALL patients

**Fig. 8:**
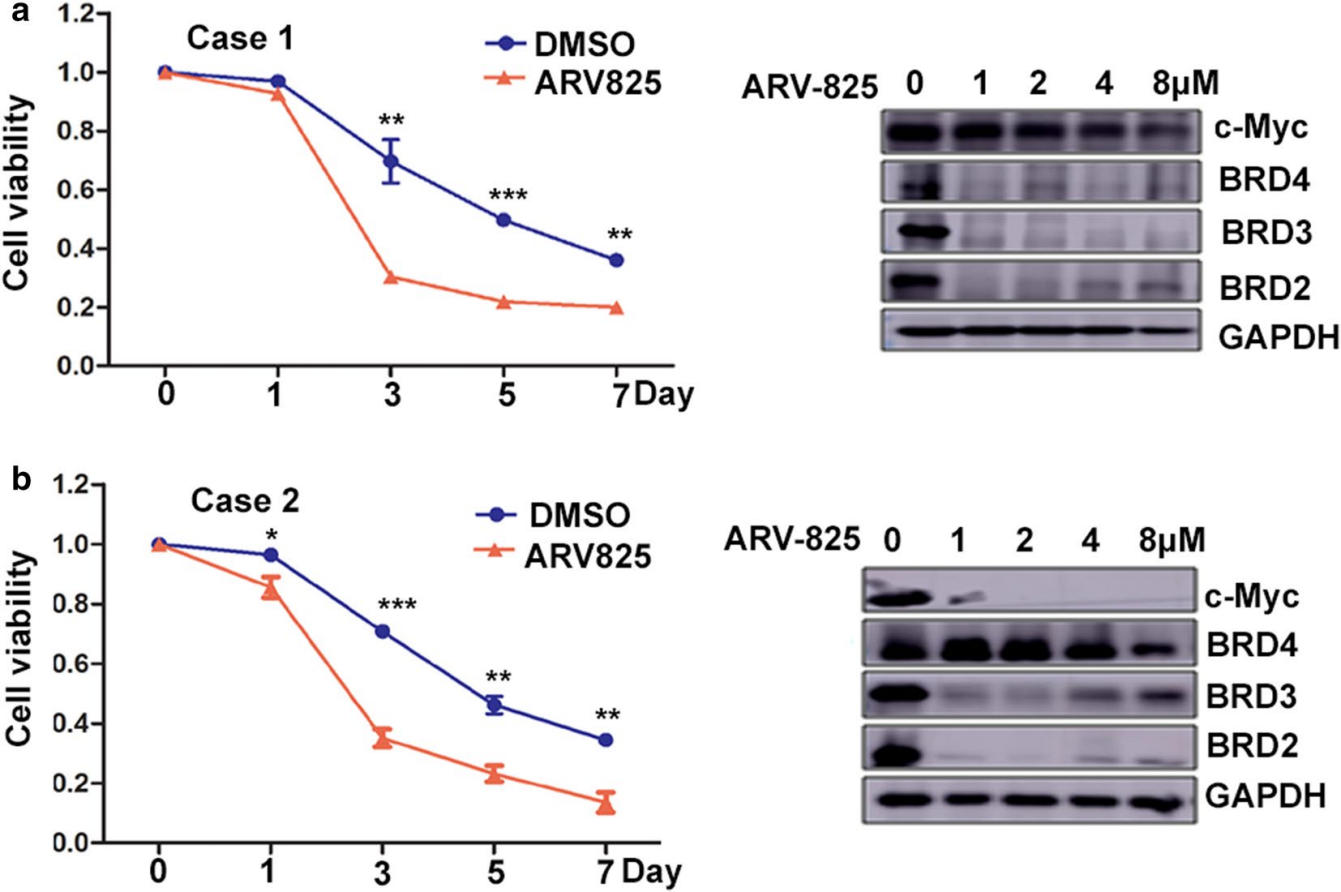
ARV-825 shows cytotoxicity in primary T-ALL cells. **a** Growth of primary T-ALL cells treatment with 1 μM ARV-825 (Case 1); ARV-825 suppressed BET and c-Myc protein expression in primary T-ALL cells from various concentration of ARV-825. **b** 8 μM ARV-825 treatment impaired growth of pediatric primary T-ALL cells (Case 2). ARV-825 suppressed BET and c-Myc protein expression in primary T-ALL cells from various concentration of ARV-825

We determined whether primary pediatric T-ALL cells were sensitive to ARV-825 treatment using two diagnostic pediatric T-ALL samples. The clinical characteristics of the patients are presented in Table 3. Both patients were primarily resistant to conventional chemotherapy, with induction failure in the CCCG2015 protocol. We treated the cells with DMSO or increasing doses of ARV-825. ARV-825 inhibited the cell growth in both pediatric T-ALL samples, and c-Myc protein levels were significantly reduced in all the ARV-825-treated cultures tested. Consistent with the hypothesis that ARV-825 inhibits c-Myc, the sensitivity of the samples to ARV-825 was correlated with the reduction in c-Myc. ARV-825 treatment also downregulated BRD2, BRD3, and BRD4 protein expression levels in primary T-ALL cells (Fig. 8), consistent with the results in cell lines. Collectively, these in vitro studies revealed the therapeutic potential of ARV-825 for pediatric T-ALL.

**Table 3:** Clinical and molecular characteristics of 2 primary pediatric T-ALL

Case	Gender	Age (y)	Karyotype comments	Fusion Gene	Mutation	Initial BM blast (%)	WBC (× 10^9^/L)	D15th BM blast (%)	D33th BM blast (%)
1	Male	3.75	88–92,XY, + X, + Y, + 1 × 2, + 2 × 2, + 3 × 2, + 4 × 2, + 5 × 2, + 6 × 2, + 7 × 2, + 8 × 2, + 9 × 2 + ……. + 22 × 2	PICALM/MLLT10	None	88	122.82	92	47
2	Female	10.25	46,XX,t (10,11) (p13,q23), add (15) (q25)/46,idem, + mar	DDX3X/MLLT10	NOTCH1 (41.2%),NRAS (80.7%)	89	35.33	82	21

### ARV-825 has a potent antitumor effect in T-ALL xenograft model

**Fig. 9:**
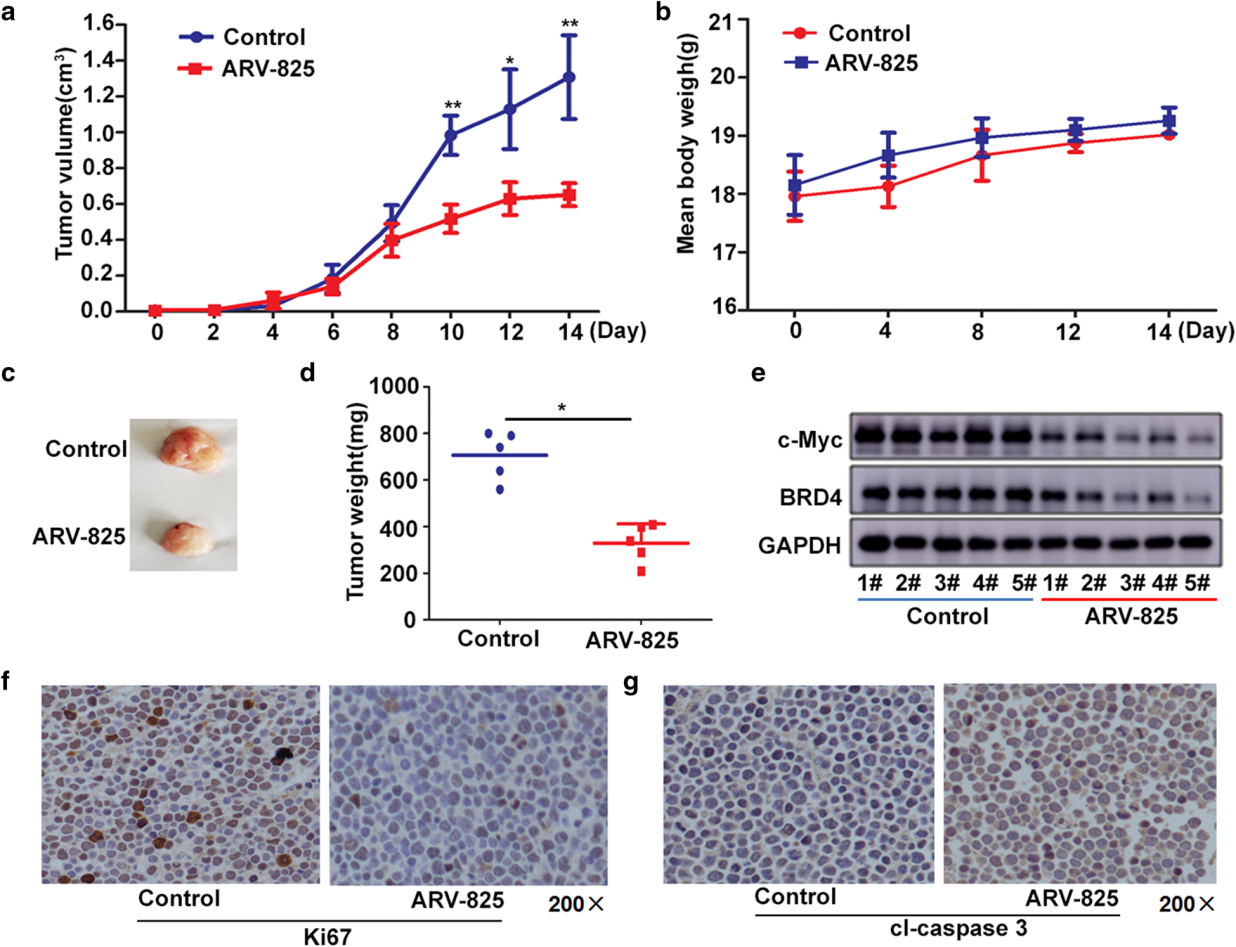
ARV-825 displays anti-tumor efficacy in the T-ALL xenograft model. Nude mice bearing CCRF xenograft tumors were treated by either 10 mg/kg ARV-825 or vehicle control intraperitoneally every day for 14 days. Data are mean ± SEM (n = 5). **a** Tumor volume was recorded every 2 days and calculated using the formula: (width × width × length)/2. **b** Mice body mass was weighed every 4 days. **c** Photograph of xenograft tumors from ARV-825- or vehicle-treated mice. **d** Tumor weight from ARV-825- or vehicle-treated mice. **e** Western blot analysis of BRD4 and c-Myc expression in tumors from ARV-825- or vehicle-treated mice. **f** IHC staining of Ki67 in xenograft tumors from ARV-825- or vehicle-treated mice. **g** IHC staining of cleaved-caspase 3 (cl-caspase 3) in xenograft tumors from ARV-825- or vehicle-treated mice

To further investigate the in vivo activity of ARV-825, we developed the pre-clinical model of T-ALL using CCRF cells. Two weeks after the cells were inoculated, 10 mg/kg ARV-825 or vehicle alone (5% Kolliphor^®^HS15) were administrated daily into the mouse. A significant reduction of tumor burden was observed in mice with ARV-825 treatment group compared with control group (Fig. 9a). No significant difference of mice body weight was observed between treatment and control group (Fig. 9b), but the xenograft tumor weight was reduced in mice receiving ARV-825 treatment (Fig. 9c, d). ARV-825 treatment downregulated the BRD4 and c-Myc protein expression in ARV-825-treated xenograft tumors (Fig. 9e), which is consistent with the in vitro results. Besides that, Ki67 positive cells were reduced in tumors from ARV-825-treated mice (Fig. 9f), while the proportion of cleaved-caspase 3 positive cells was increased in tumors from ARV-825-treated mice (Fig. 9g), indicating the dysregulation of proliferation and apoptosis. These observations suggest that ARV-825 can effectively suppress tumor growth in T-ALL xenograft model.

## Discussion

T-ALLs are aggressive proliferations of transformed T-cell progenitors. Although intensification of chemotherapeutic schedules has greatly improved the prognosis of T-ALL in the past 10 years, around 30% of cases still relapse within the first 2 years following diagnosis [26]. A detailed understanding of the T-ALL oncogenic networks and the escape pathways involved in acquiring chemoresistance is therefore required.

BRD4 plays important roles in a number of cancer types, including prostate cancer, lung carcinoma, and melanoma, as well as hematologic malignancies [6–9, 12, 27]. However, the biological significance of BRD4 in T-ALL is less known. We initially examined BRD4 expression and found that BRD4 was the top-ranked gene in T-ALL cell lines relative to other types of cancer cell lines in CCLE samples. Additionally, compared with healthy samples, T-ALL samples showed higher BRD4 mRNA expression levels. RNA-seq analysis in the current study accordingly showed that BRD4 was commonly expressed in T-ALL patients, while survival analysis indicated that BRD4 was a poor prognostic marker for pediatric T-ALL patients. Although BRD4 expression was not a significant independent factor affecting survival in pediatric T-ALL patients, according to multiple logistic regression analyses, patients with high expression had a four-fold greater risk of death compared with patients with low expression levels. BRD4 has been shown to cluster at super-enhancer regions involved in the control of pivotal oncogenes, such as c-Myc, Bcl-xl, and Bcl-2 [14, 28–30], suggesting that it might be a target providing an alternative strategy to improve outcomes for T-ALL patients.

There are currently numerous BRD4 inhibitors, such as JQ1 and OTX015. Indeed, both preclinical and clinical studies have shown that the effects of these inhibitors are largely cytostatic, with apoptosis limited to a few cell lines and tumors from phase I patients. These agents also showed limited clinical activity and a general lack of sustained transcriptional inhibition of their targets, which could significantly limit their potential clinical benefits [10, 11, 30]. One strategy to improve BRD4 inhibition that has seen renewed interest in recent years involves designing irreversible/covalent inhibitors able to achieve the desired pharmacological effect at lower drug concentrations. ARV-825 is a novel BRD4 degrader that exerts superior lethal activity compared with BET inhibitors in many tumors [15–17, 31, 32]. Saenz et al. demonstrated that ARV-825 mediated BET protein degradation and was more effective than existing BET inhibitors at blocking BET protein transcriptional function in lymphoma and acute myelocytic leukemia [14]. However, its cytotoxicity in T-ALL was not explored. Our results showed that ARV-825 could effectively attenuate T-ALL cell growth in vitro and in vivo by suppressing cell proliferation and accelerating apoptosis. The cytotoxicity of ARV-825 was superior to that of dBET1 (another PROTAC BRD4 inhibitors), in addition to conventional inhibitors. Meanwhile, ARV-825 also increased cleaved PARP and cleaved-caspase 3 in Jurkat, Molt4, and 6 T-CEM cells, consistent with increased apoptosis. Our data also indicated that triggering cell cycle arrest, resulting in more cells in the resting state, was key for treatment with ARV-825. These results were consistent with previous observations on solid tumors and acute myeloid leukemia (AML) [14–16].

BET proteins, including BRD2, BRD3, BRD4, and BRDT, are epigenome readers known to associate with acetylated chromatin and transcriptional regulation. Our findings confirmed that not only BRD4, but also BRD2 and BRD3 protein levels were reduced after ARV-825 treatment. Similar findings were reported in other studies [11, 16]. This phenomenon could be due to OTX015 in ARV-825, which can bind to all BET family members because of the high homology in domains among BET family members [31]. Compared with OTX015, ARV-825 is a PROTAC that targets BRD4 and BET family proteins for CRBN-mediated proteasomal degradation [14], potentially resulting in profound depletion of BET proteins. Moreover, our results showed that ARV-825 was more potent in degrading BRD2 than BRD4 in Jurkat and CCRF cells. OTX015 and ARV 825 significantly degraded BRD 2 and BRD4 [11, 16], while the degradation of BRD4 was correlated to CRBN expression in MM cells [2]. So we speculated this result may be due to the low level of CRBN in Jurkat and CCRF cells. Further investigations are required to determine the different influences and intrinsic mechanisms of BET inhibitors on BET family proteins.

Previous studies showed that CRBN expression level was a predictive marker for ARV-825 efficacy [33]. The current findings confirmed that CRBN expression played an essential role in the sensitivity of T-ALL cells to ARV-825, given that shRNA-mediated knockdown or overexpression of CRBN reduced and increased their sensitivity to ARV-825, respectively. Surprisingly, we did not observe positively relationship between ARV-825 and CRBN expression among T-ALL cell lines. CCRF cells with lowest CRBN were most sensitive to ARV-825. The possible explanation was that the potency of ARV-825 on T-ALL cell viability relied on its capability of degradation of BRD proteins and suppression of the downstream c-Myc or other target genes expression in different cells, which should be proofed in near future. Additionally, blocking the proteasome by MG132 remarkably inhibited the ARV-825-induced BRDs degradation. These results indicated that the enhanced anti-leukemic effect of ARV-825 was closely related to CRBN, consistent with results in other tumors [18, 34].

C-Myc is transcriptionally regulated by super-enhancers in several hematological malignancies. Super-enhancers comprise broad areas of active open chromatin marked by H3K27 acetylation. BRD4 densely occupies super-enhancers and its inhibition thus reduces c-Myc transcription, causing an anti-tumor effect [11, 13, 29, 30]. In the current study, inhibition of BET proteins by disturbing H3K27 acetylation led to the downregulation of multiple genes (*CARMIL2*, *IGLL1*, *MYC*, *ADAM33*, and *PTPRF*) and the upregulation of histones responsible for the nucleosome structure, including *H1-0*, *H1-10*, *H2BC20P*, *H3-3B*, and *H1-2*. Interestingly, GSEA demonstrated that BRD4 depletion following ARV-825 treatment reduced Myc-pathway genes, according to RNA-seq analysis, which was further verified by western blot analysis in cell lines and primary T-ALL cells. Similar results were obtained in AML and multiple myeloma, which depended on the BRD4-Myc axis to inhibit cell proliferation in vitro and in vivo through regulation by ARV-825 [6, 14, 16]. Previous studies also showed that samples from patients with induction failure and relapse resistant to γ-secretase inhibitor treatment remained sensitive to BET inhibitors, depending on c-Myc reduction [13], and suppression of Myc activity led to T-ALL remission [35]. Similarly, the two pediatric T-ALL patients in the present study who were primarily resistant to conventional chemotherapy in the CCCL2015 protocol remained sensitive to ARV-825 with c-Myc downregulation. Collectively, these results reveal a crucial role for c-Myc in drug resistance in leukemia.

The limitation of current study was that the sample size for primary T-ALL and healthy donor were small, which would have weakened the findings. More studies are therefore needed to validate our conclusions to allow this drug to be entered into clinical trials to benefit more patients.

## Conclusions

The results of this study demonstrated that ARV-825 had potent anti-tumor activity in T-ALL cell lines and primary pediatric T-ALL samples. ARV-825 exerts its effects by degrading BET proteins, leading to c-Myc suppression. These findings suggest that ARV-825 may represent a promising therapeutic approach for T-ALL.

## Supplementary Information

**Additional file 1:** **Table S1**. Clinical and molecular characteristic of pediatric T-ALL patients in this study.

**Additional file 2:** **Figure S1**. ARV-825 induces strong degradation in a dose-dependent manner.

**Additional file 3: Table S2**. GSEA_HALLMARK_MYC_TARGETS gene set in 6T-CEM.

**Additional file 4: Table S3.** GSEA_HALLMARK_MYC_TARGETS gene set in Jurkat.

## Data Availability

The datasets used and/or analyzed during the current study are available from the corresponding author on reasonable request.

## Abbreviations

T-ALL: T-cell acute lymphoblastic leukemia
BET: Bromodomain and extra terminal
BRD4: Bromodomain-containing protein 4
CCK-8: Cell Counting Kit-8
IC50: Half-maximal inhibitory concentration
PBS: Phosphate-buffered saline
PCR: Polymerase chain reaction
shRNA: Short hairpin RNA
