# Supplementary material for: BRD4 PROTAC degrader ARV-825 inhibits T-cell acute lymphoblastic leukemia by targeting 'Undruggable' Myc-pathway genes
Source: Cancer Cell Int. 2021 Apr 22;21:230. doi: 10.1186/s12935-021-01908-w (PMC8061034; doi:10.1186/s12935-021-01908-w)
Supplement: Supplementary file 1 — Additional file 1: Table S1. Clinical and molecular characteristic of pediatric T-ALL patients in this study. [file 12935_2021_1908_MOESM1_ESM.docx]

Additional file 1: Table S1. Clinical and molecular characteristic of pediatric T-ALL patients in this study.

|  | Pediatric T-ALL(n=46) |
| --- | --- |
| Gender, n (%) |  |
| Male | 38(83%) |
| Female | 8(17%) |
| Age at diagnosis,years,median (range) | 7.65(1.2-12.2) |
| Initial WBC,(x109/L), median (range) | 147.31(4.14-693.82) |
| Hemoglobin, g/L, median (range) | 102(49-152) |
| Platelet, (x109/L), median (range) | 78(10-263) |
| Genetic subtypes, n (%) |  |
| SIL/TAL1 | 11(24) |
| HOX11+ | 3(7) |
| MLL/EAF9 | 1(2) |
| MLL/ENL | 1(2) |
| TCRβ Rearranged | 1(2) |
| Normal | 29(63) |
| Karyotype, n (%) |  |
| normal | 16(35) |
| Other structural abnormal | 11(24) |
| numerical abnormal | 3(6) |
| Failure or Missing | 16(35) |
| Prednisone Response, n(%) |  |
| Poor | 28(61) |
| Good | 18(39) |
| D15th BM blast, n (%) |  |
| M1 | 17(37) |
| M2 | 10(22) |
| M3 | 19(41) |
| Day 33rd BM blast, n (%) |  |
| M1 | 40(87) |
| M2 | 6(13) |
| Week 12th BM blast, n (%) |  |
| M1 | 43(93) |
| M2 | 3(7) |
| Risk group, n (%) |  |
| Intermediate risk | 7(15) |
| High risk | 39(85) |
