# Supplementary material for: BRD4 PROTAC degrader ARV-825 inhibits T-cell acute lymphoblastic leukemia by targeting 'Undruggable' Myc-pathway genes
Source: Cancer Cell Int. 2021 Apr 22;21:230. doi: 10.1186/s12935-021-01908-w (PMC8061034; doi:10.1186/s12935-021-01908-w)
Supplement: Supplementary file 2 — Additional file 2: Figure S1. ARV-825 induces strong degradation in a dose-dependent manner. [file 12935_2021_1908_MOESM2_ESM.docx]

**Additional file 2: Figure S1. ARV-825 induces strong degradation in a dose-dependent manner.**

**
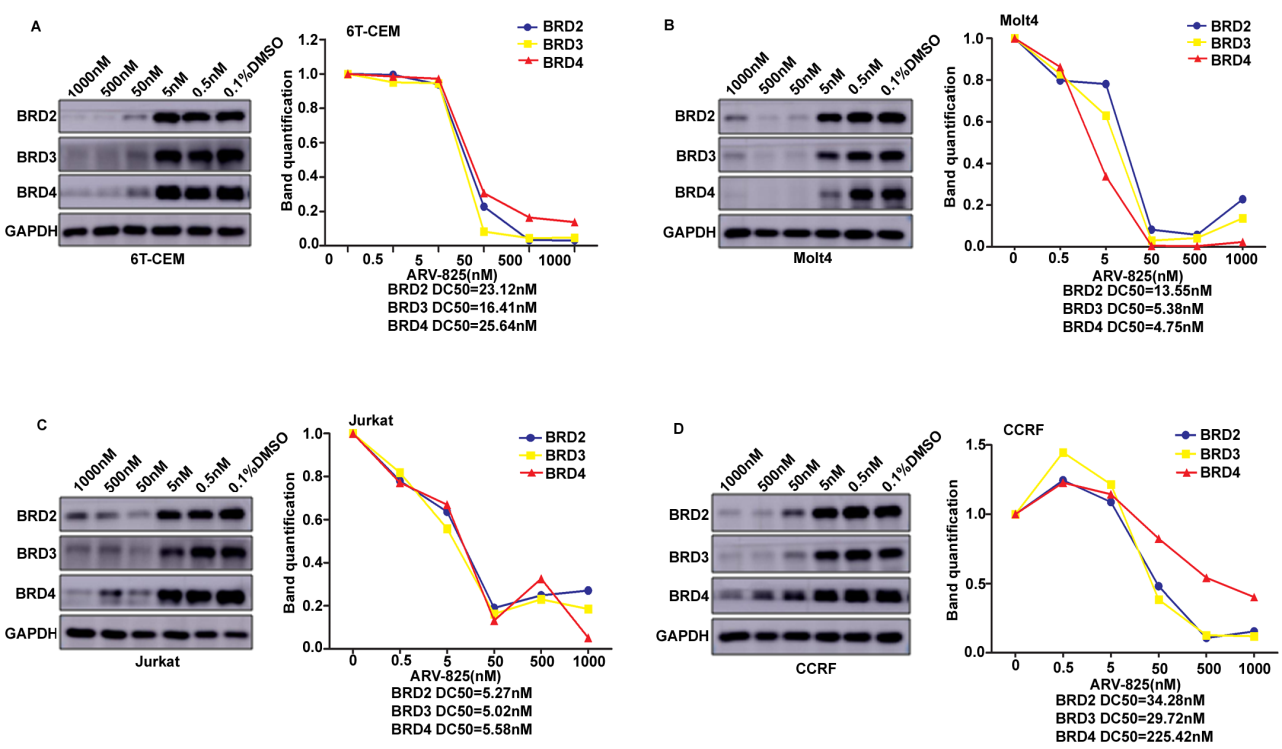
**

(A, B, C and D, left): Western-blot analysis of BRD2, BRD3, BRD4 and GAPDH after treatment of T-ALL cells (6T-CEM, Molt4, Jurkat and CCRF) with six different concentrations of ARV-825 at 48h before harvesting; (A, B, C and D, right): Quantification of protein levels relative to DMSO control in T-ALL cells after treatment with different concentration of ARV-825 and DC_50_ values.
